# Evaluation of Shear Performance of Integrated GFRP Stirrup Systems in Reinforced Concrete Beams

**DOI:** 10.3390/polym18080921

**Published:** 2026-04-09

**Authors:** Saruhan Kartal, Uğur Gündoğan, İlker Kalkan, Turki S. Alahmari, Abderrahim Lakhouit, Akin Duvan

**Affiliations:** 1 Department of Civil Engineering, Faculty of Engineering and Natural Sciences, Kirikkale University, Kirikkale 71451, Turkey; 2 Institute of Science, Kirikkale University, Kirikkale 71451, Turkey; 3 Department of Civil Engineering, Faculty of Engineering, University of Tabuk, Tabuk 71491, Saudi Arabia; 4 Department of Civil Engineering, Engineering Faculty, Karamanoglu Mehmetbey University, Karaman 70200, Turkey

**Keywords:** GFRP RC beams, shear behavior, stirrup configuration, transverse reinforcement, load capacity

## Abstract

This study investigates the shear behavior of glass fiber-reinforced polymer (GFRP)-reinforced concrete (RC) beams to address challenges associated with their low elastic modulus, absence of yielding, and reduced stirrup efficiency in bending regions. GFRP bars are increasingly adopted as an alternative to steel due to their superior corrosion resistance, durability, and cost-effectiveness. This study focuses on the effects of stirrup type, stirrup spacing, and shear span-to-effective depth ratio on the structural performance of GFRP RC beams. Twelve full-scale beams were tested under four-point bending, incorporating three GFRP shear reinforcement configurations: fabricated closed stirrups, integrated straight bar systems, and discrete vertical bars. Experimental observations were analyzed in terms of failure modes, load-carrying capacity, energy absorption, and deformation characteristics. Results indicate that fabricated F-type stirrups provide the highest shear performance, though their effectiveness is limited by premature rupture at bending points. Site-integrated S- and T-type configurations offer practical alternatives, maintaining structural integrity while mitigating bend-related stress concentrations, but with slightly lower energy absorption and load capacity. Increasing stirrup spacing significantly reduces shear resistance and shifts failure from flexural to shear-dominated modes. Comparisons with widely used design codes and analytical models show that CSA S806-12 provisions offer the most reliable predictions, while other guidelines tend to over- or underestimate shear capacity depending on configuration and a/d ratio. The study highlights the importance of optimizing stirrup type and spacing to enhance the shear performance of GFRP RC beams. Findings provide valuable insights for improving current design methodologies, offering guidance for engineers seeking durable, corrosion-resistant alternatives to steel reinforcement in aggressive environments. This research demonstrates that innovative site-integrated stirrup configurations can bridge practical fabrication constraints without compromising overall shear performance, promoting more efficient and resilient GFRP RC structures.

## 1. Introduction

The strategic replacement of conventional steel with Fiber-Reinforced Polymer (FRP) reinforcement has emerged as a definitive solution for mitigating the multi-billion dollar economic impact of corrosion-induced structural degradation in maritime and chemically aggressive environments. Among the various fiber compositions, Glass Fiber-Reinforced Polymer (GFRP) is predominantly favored due to its superior cost-efficiency and high tensile strength [1,2,3,4,5]. However, unlike the ductile and isotropic nature of steel, GFRP exhibits an anisotropic, linear-elastic behavior until failure. A critical design challenge lies in its low elastic modulus (*E_f_*), which is approximately one-fourth that of steel. This lower axial stiffness leads to wider diagonal cracks and a reduced neutral axis depth, significantly diminishing the concrete’s natural shear-transfer mechanisms, such as aggregate interlock and the compression zone’s contribution (*V_c_*) [6,7].

Recent advancements in structural engineering have seen a global trend toward innovative technologies and sophisticated optimization strategies to overcome such material limitations. Recent research has increasingly focused on maximizing structural reliability through multi-parameter evaluation, such as the use of genetic algorithms for optimizing complex members like prestressed stayed steel columns [8]. Following this shift toward material synergy, hybrid FRP-steel systems have emerged to balance composite durability with steel ductility, though their performance remains highly sensitive to reinforcement proportions and energy-based ductility limits [9]. Furthermore, the development of durable components—such as hybrid shear walls with replaceable dampers analyzed via strut-and-tie models [10]—underscores a shift toward repairable and optimized engineering solutions. Despite these technological advancements, the practical application of GFRP-RC systems still faces fundamental analytical challenges.

A critical review of the existing literature reveals that the shear resistance of GFRP-RC beams without transverse reinforcement is a function of complex parameters, including the reinforcement ratio and the shear span-to-effective depth ratio (a/d). Detailed assessments of large-scale experimental databases by Ali et al. [1] (*n* = 510), El Zareef et al. [11] (*n* = 386), and Alguhi and Tomlinson [12] (*n* = 147) have consistently demonstrated that while the CSA S806-12 [2] code offers relatively consistent and physically representative shear strength predictions for slender beams (a/d > 2.5), the ACI 440.1R-15 [4] guidelines remain excessively conservative. Furthermore, the accuracy of these codes is highly sensitive to the member depth and a/d ratio. For deep beam configurations (a/d < 2.5), Mukhtar and Deifalla [3] identified that current codes fail to adequately account for the transition from beam action to arch action, a finding corroborated by El Zareef et al. [11], who noted significant deviations for depths exceeding 300 mm. To bridge these gaps, researchers have utilized advanced methodologies, including multivariate nonlinear regression [1], numerical parametric investigations into concrete strength [5], and artificial intelligence-based approaches like Generalized Regression Neural Networks (GRNN) [13], to propose more refined predictive expressions. Moreover, Kara and Ashour [14] emphasized that the “size effect” in CFRP-RC beams remains a critical factor that is not yet fully integrated into standard design formulations.

The efficacy of GFRP stirrups in shear-critical members is predominantly limited by the “bend effect,” which remains a major structural impediment. As corroborated by Liang et al. [15] and Al-Hamrani and Alnahhal [16], the thermomechanical processes involved in creating factory-prefabricated closed-loop geometries induce significant fiber misalignment and severe stress concentrations at the 90-degree bend zones. These phenomena trigger a catastrophic localized strength reduction of approximately 40–60% relative to the longitudinal axis of straight counterparts [17], thereby precluding the full mobilization of the polymer’s theoretical tensile capacity. To compensate for this localized vulnerability, international design codes prescribe highly restrictive effective strain thresholds; specifically, ACI 440.1R-15 [4], CSA S806-12 [2], and JSCE-97 [18] recommend limit strain values of 0.004, 0.005, and 0.0025, respectively.

However, the validity of these thresholds remains a subject of intense academic debate. Said et al. [19] reported that increasing the GFRP stirrup ratio and concrete compressive strength can enhance shear capacity by up to 104%, while Al-Hamrani and Alnahhal [16] observed a 20% capacity increase by optimizing stirrup spacing from 250 mm to 170 mm. Issa et al. [20] further noted that stirrup strain values are non-uniformly distributed and heavily influenced by the a/d ratio, which governs the failure mode transition from diagonal tension to compression-tension failure. Despite these potential gains, the influence of the “size effect” cannot be entirely eliminated in large-scale members, as highlighted by Jumaa and Yousif [21].

To mitigate the ‘bend effect’ [15,16,17] and overcome the conservative design thresholds imposed by current codes [2,4,18], researchers have explored hybrid steel-FRP systems [22], carbon fabric configurations [17], and integrated bars [23], with Liang et al. [15] even suggesting the use of FRP grids to avoid the weaknesses of bending points. Beyond structural performance, the widespread industrial implementation of GFRP stirrups is hindered by logistical and economic barriers. Unlike steel reinforcement, GFRP bars cannot be bent on-site due to their nature. Consequently, any variation in structural dimensions requires size-specific factory molds for stirrup production. This dependency on custom tooling increases manufacturing complexity, production costs, and lead times, creating a significant logistical bottleneck. While preliminary studies, such as those by Duic et al. [23], have explored integrated reinforcement systems using straight rebars, there remains a notable lack of comparative research evaluating the structural reliability of site-assembled discrete bar configurations versus traditional prefabricated closed stirrups.

To overcome the necessity for size-specific molds and the associated manufacturing overheads, this research explores innovative, site-adaptable reinforcement configurations. The novelty of the present investigation resides in the systematic development and performance evaluation of innovative “S-Type” and “T-Type” integrated GFRP transverse reinforcement architectures, synthesized from discrete straight bars. By analyzing 12 full-scale RC beams, this study interrogates the viability of these site-adaptable configurations as a robust mechanism to circumvent the logistical manufacturing constraints and the “bend effect” inherent in conventional closed stirrups, without jeopardizing the global shear integrity of the structural members. The experimental framework rigorously examines the multi-parametric interplay between reinforcement topology, stirrup spacing, and the shear span-to-depth (a/d) ratio. Furthermore, the empirical findings are benchmarked against four prominent international design standards (ACI 440.1R-15 [4], CSA S806-12 [2], ISIS-M03 [24], and CNR-DT 203 [25]). Ultimately, this research seeks to establish the fundamental scientific framework necessary to transition from labor-intensive, factory-dependent components to high-performance, site-integrated GFRP shear reinforcement systems that optimize structural safety and economic feasibility. This study uniquely proposes and experimentally evaluates site-integrated S-Type and T-Type GFRP stirrup configurations, overcoming the bend effect of conventional closed fabricated stirrups while preserving shear performance.

## 2. Experimental Study

### 2.1. Test Specimens and Material Mechanical Properties

The experimental study involved the testing of 12 full-scale GFRP-RC beams systematically designed to evaluate the shear performance of innovative transverse reinforcement architectures. All specimens featured a uniform rectangular cross-section of 220 mm in width and 300 mm in height. A critical design objective was to ensure a shear-dominant failure mechanism and strictly preclude premature flexural collapse. Consequently, the specimens were designed as heavily over-reinforced sections, with a longitudinal reinforcement ratio (*ρ_f_*) established at approximately 4.26 times the balanced reinforcement ratio (*ρ_fb_*). This configuration was achieved by utilizing six Ø12 mm GFRP bars in the tension zone and two Ø12 mm GFRP bars in the compression zone. Six of these specimens are 3000 mm with a net span of 2700 mm, and the remaining six are 2300 mm with a net span of 2000 mm in length. The test matrix was structured around three primary independent variables: transverse reinforcement type (F, S, and T types), stirrup spacing (*s*), and the shear span-to-effective depth ratio (a/d). The beam lengths of 3000 mm and 2300 mm were selected to correspond to two different a/d ratios, namely approximately 3.8 and 2.6, respectively.

**Figure 1:**
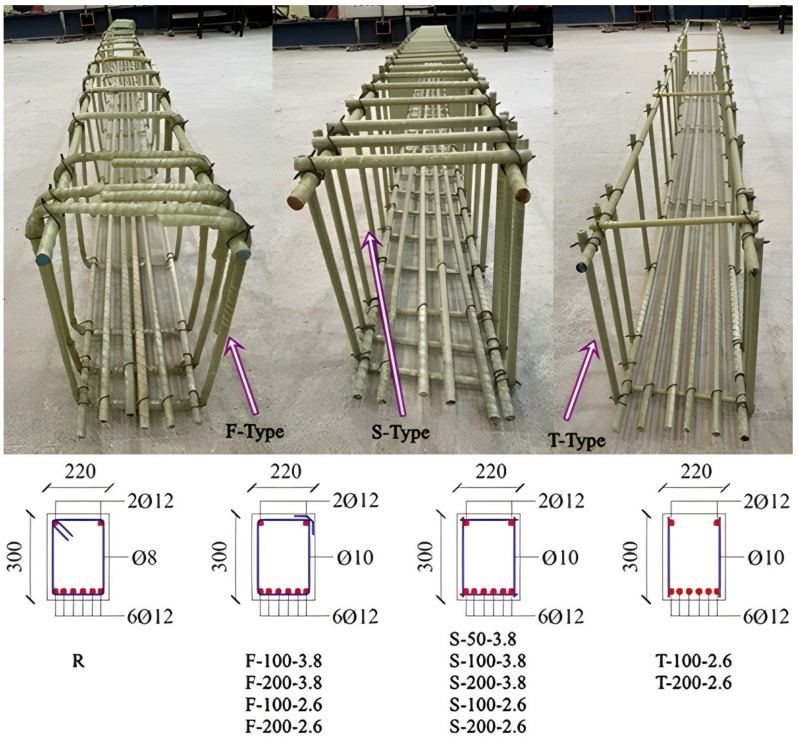
Types of GFRP stirrups.

A central novelty of this investigation resides in the comparative performance evaluation of traditional prefabricated stirrups against innovative site-integrated discrete bar architectures. Three distinct transverse reinforcement configurations (Figure 1), all utilizing Ø10 mm GFRP bars, were systematically investigated: F-Type, representing the industry-standard factory-fabricated closed-loop GFRP stirrups with 90-degree bent corners; S-Type, an innovative site-integrated architecture synthesized from four discrete straight GFRP rebars assembled to form a closed perimeter; and T-Type, a simplified system consisting of two discrete vertical GFRP bars. The S-Type configuration, in particular, was engineered as a robust mechanism to circumvent the intrinsic micro-structural damage and the catastrophic localized strength reduction, while the T-Type serves as a critical benchmark for assessing the fundamental shear-transfer efficiency of discrete vertical reinforcement.

**Figure 2:**
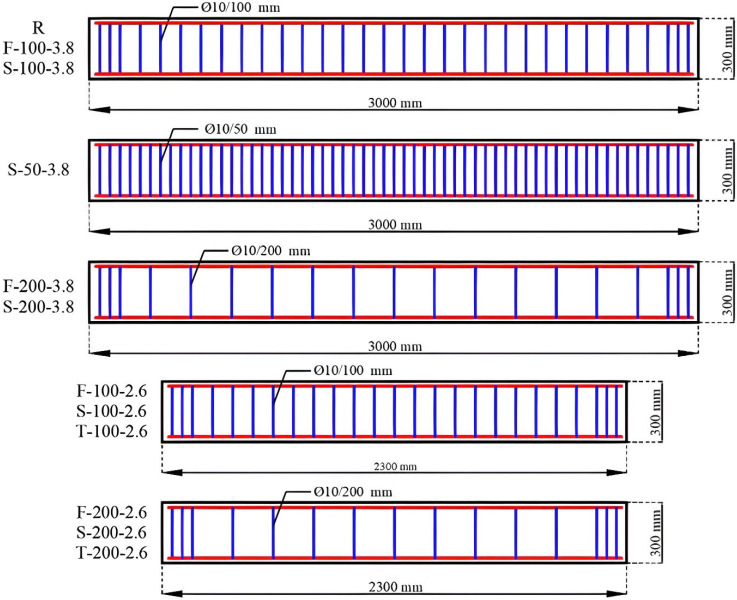
Reinforcement details of RC beams.

The spacing of stirrups was set at 100 mm and 200 mm. To account for the possibility of GFRP-reinforced test specimens not reaching their bending capacity, a test program included one specimen reinforced with steel stirrups (Ø8/100 mm) and having an a/d ratio of 3.8, as well as one specimen reinforced with S-type stirrups (Ø10/50 mm) and the same a/d ratio of 3.8. Details of the test specimens are given in Table 1, while the reinforcement details are presented in Figure 2.

In the nomenclature of the specimens, the first capital letter indicates the type of stirrup used in the specimen. F-type, S-type and T-type are denoted as “F”, “S” and “T”, respectively. Furthermore, the specimen with steel stirrups is indicated by “R”. The numbers “50”, “100” and “200” represent the spacing of stirrups in millimeters, while the numbers “3.8” and “2.6” symbolize the beam’s a/d ratios.

**Figure 3:**
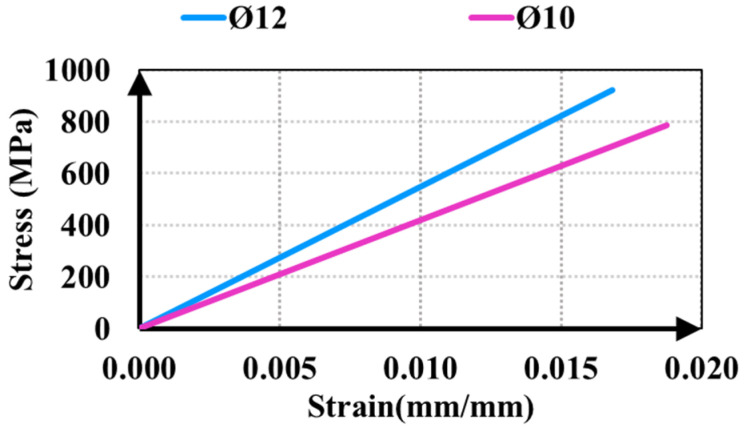
Stress strain curves of the GFRP reinforcement.

All test specimens were prepared by casting the concrete in a single pour. The concrete was a ready-mix structural concrete designed with a water-cement ratio of 0.55 and a maximum aggregate size of 16 mm. This specific aggregate size was selected to ensure a consistent and reliable aggregate interlock mechanism throughout the shear-critical zones. The average compressive strength of 20 cylindrical specimens with dimensions of 150 × 300 mm, taken from the concrete, is 21.92 MPa. In the study, Ø12 GFRP longitudinal reinforcement was used, while Ø10 reinforcement was preferred for stirrups. A horizontal test setup was established to determine the mechanical properties of GFRP reinforcement. The mechanical properties of GFRP reinforcement are presented in Table 2 based on the results of axial tensile tests. Furthermore, the stress–strain relationships for the GFRP reinforcement are illustrated in Figure 3.

The average concrete compressive strength was measured as 21.92 MPa. This value was intentionally kept at a moderate level, while the specimens were designed with high flexural capacity to ensure a shear-critical behavior. This strategic experimental design was adopted to ensure that the concrete’s inherent shear strength (*V_c_*), which is primarily governed by aggregate interlock and the integrity of the compression zone, would not be sufficient to resist the applied shear loads on its own. By preventing premature flexural failure and limiting the concrete’s contribution, the study was able to effectively isolate and accurately evaluate the contribution of the different GFRP stirrup configurations to the total shear capacity. While it is acknowledged that higher concrete strength would typically enhance the shear resistance—as emphasized by the design equations of ACI 440.1R-15 [4] and CSA S806-12 [2]—maintaining a constant and moderate strength was essential to fulfill the research objective of evaluating the reinforcement systems’ performance under critical shear conditions.

### 2.2. Test Setup

**Figure 4:**
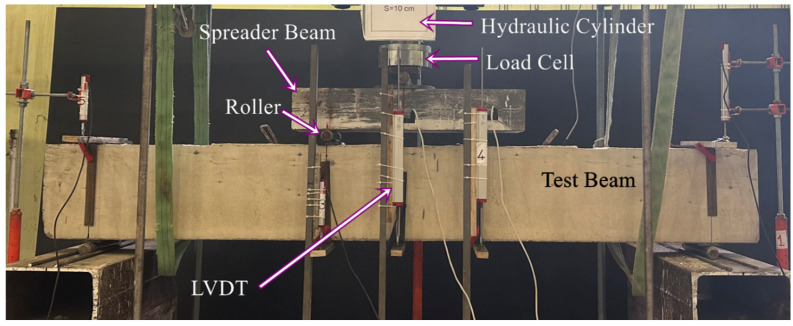
Test setup.

Four-point bending tests were conducted on the steel frame located at Kırıkkale University Structural Mechanics Laboratory. The load was applied at two points through the spreader beam, each point located 250 mm away from the midpoint of the beam. Six potentiometers were employed for vertical displacement measurements. These measurements were taken from the front and rear surfaces of the midpoint of the beam, as well as from both supports and both load points. The test setup is illustrated in Figure 4.

## 3. Results and Discussion

### 3.1. Failure Modes

As previously established in the experimental design, a high longitudinal reinforcement ratio (*ρ_f_*/*ρ_fb_* = 4.26) was strategically implemented to ensure elevated flexural capacity, thereby effectively localizing structural distress within the shear span. Under these conditions, the majority of the specimens exhibited shear-critical failure modes, which allowed for a comprehensive comparative analysis of the proposed innovative stirrup configurations and conventional reinforcement architectures.

**Figure 5:**
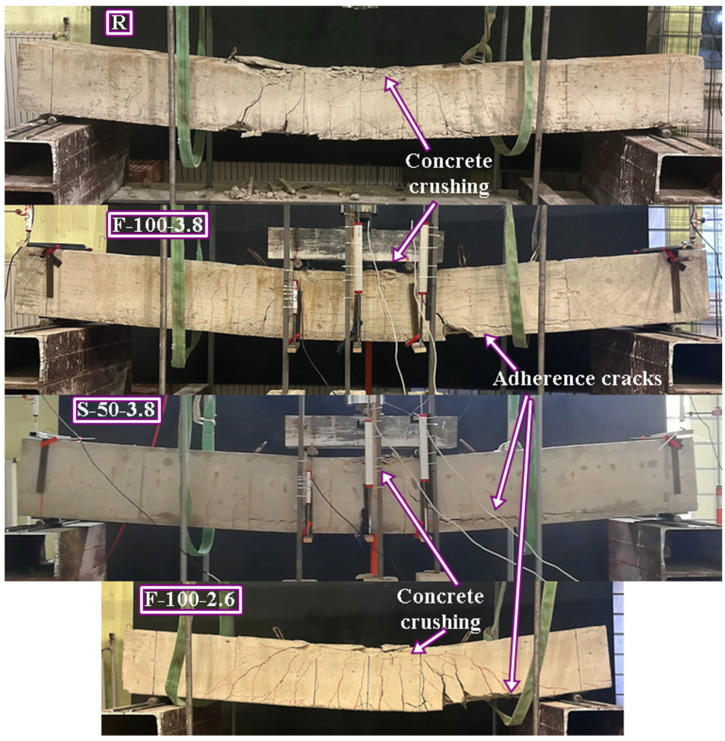
Failure modes of RC beams (observed flexural failure modes).

The specimens that successfully reached their theoretical flexural capacities are illustrated in Figure 5. This group includes the reference beam (R) and specimens F-100-2.6, F-100-3.8, and S-50-3.8. In these cases, the transverse reinforcement provided sufficient confinement and shear resistance to allow the mobilization of the full flexural strength of the concrete and longitudinal GFRP bars. Mechanically, the reduced stirrup spacing (50–100 mm) in these specimens effectively limited diagonal crack widths and maintained the integrity of the concrete compression zone until flexural failure occurred. Additionally, horizontal bond cracks were observed along the longitudinal reinforcement level in these high-capacity specimens. This phenomenon is attributed to the high reinforcement ratio, which induces elevated interfacial bond stresses; however, the adequate confinement provided by the dense stirrup arrangement prevented these cracks from progressing into splitting failure.

**Figure 6:**
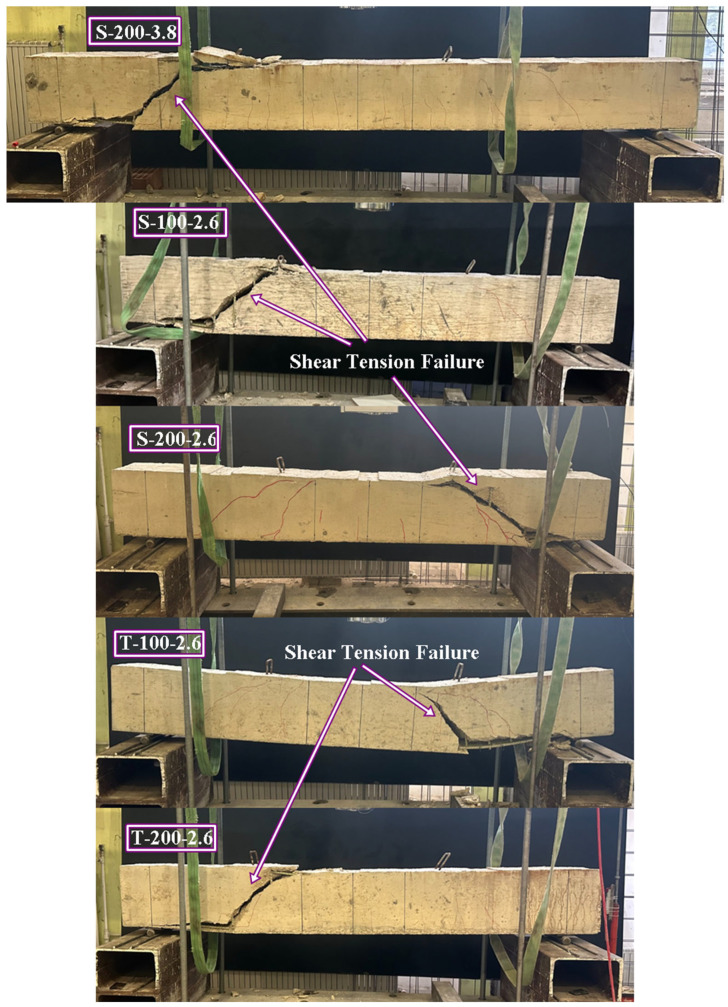
Failure modes of RC beams (observed shear tension failure modes).

**Figure 7:**
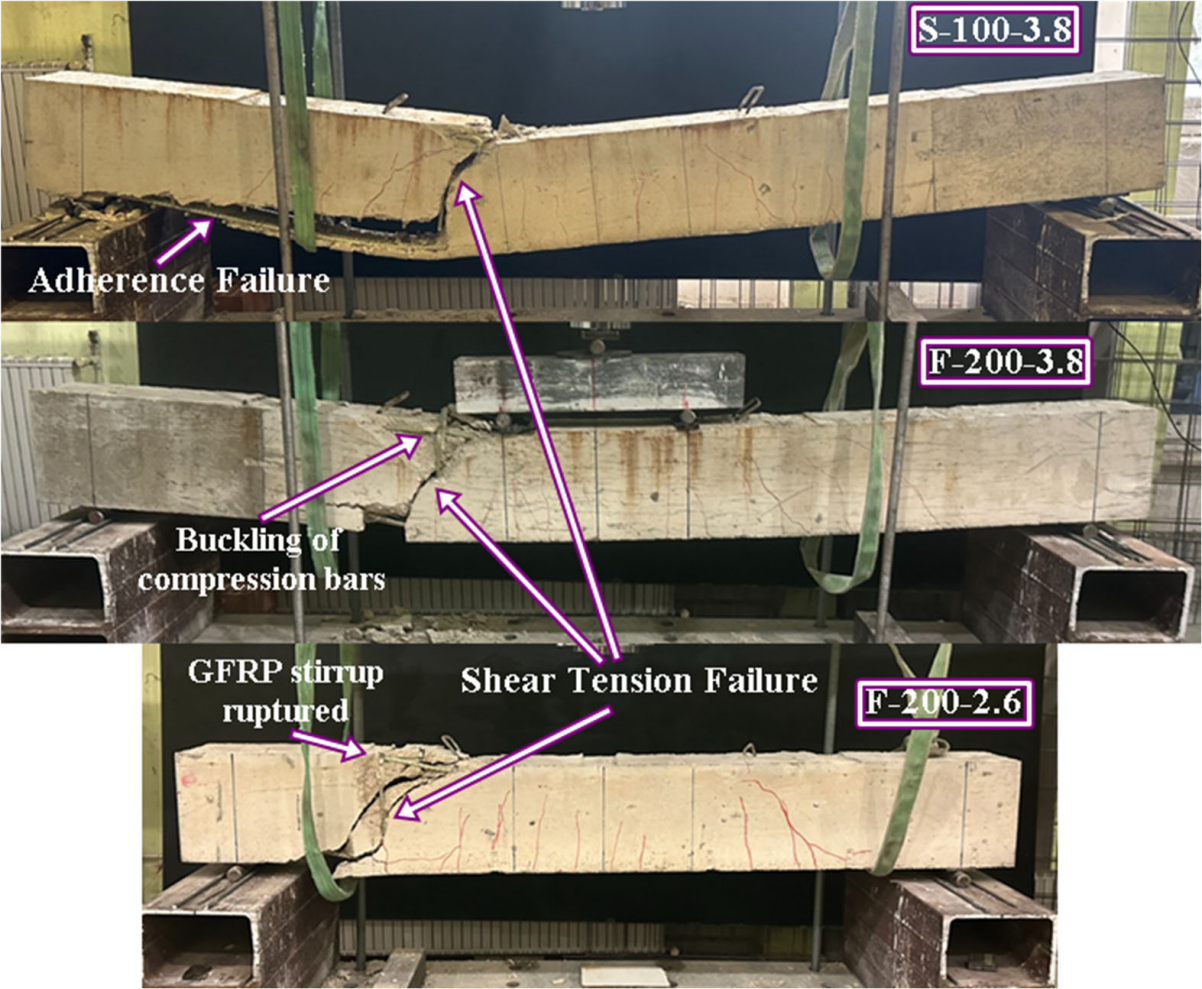
Failure modes of RC beams (observed hybrid modes).

Conversely, the specimens that failed prematurely due to diagonal shear tension failure are categorized and presented in Figure 6 and Figure 7. This behavior was predominantly observed in beams reinforced with S-type and T-type stirrups at larger spacings (100 mm and 200 mm). A particularly complex failure sequence, depicted in Figure 7, was observed in specimen F-200-3.8. In this instance, the diagonal tension failure occurred simultaneously with the mechanical ‘unhooking’ (opening) of the first F-type stirrup situated immediately to the left of the loading point. This localized loss of confinement disrupted internal stress distribution, directly triggering the buckling of the compression reinforcement. A more catastrophic response was observed in F-200-2.6 (Figure 7), where the stirrup experienced a brittle rupture at the 90-degree bend zone—a direct consequence of the ‘bend effect’—leading to a sudden loss of confinement and rapid shear collapse. Furthermore, as illustrated in Figure 7, specimen S-100-3.8 exhibited a hybrid failure mode characterized by simultaneous diagonal tension and adherence (bond) failure. In this case, the S-type stirrups underwent localized slipping from the longitudinal bars between the critical shear crack and the support. From a mechanical perspective, this suggests that while the S-type configuration offers an innovative site-adaptable alternative, its effectiveness is sensitive to the bond-slip mechanism and the structural integrity of the interlocking segments when subjected to high shear-induced transverse stresses.

### 3.2. Load–Deflection & Stiffness Relationship

**Figure 8:**
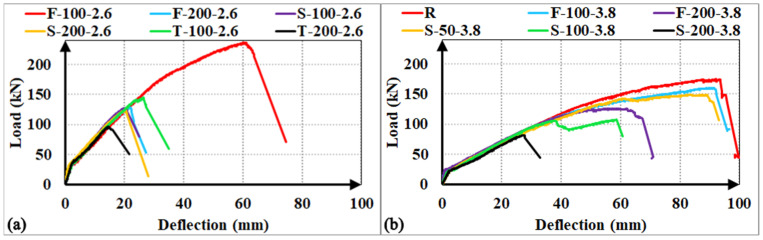
Load–deflection curves of the RC beams (**a**) shear span-to-depth ratio a/d = 2.6; (**b**) a/d = 3.8.

The experimental load-deflection responses for all investigated specimens are illustrated in Figure 8. Given the uniform cross-sectional dimensions and identical longitudinal reinforcement ratios across the matrix, all specimens exhibited nearly identical initial cracking loads and pre-cracking stiffness in each group. Upon reaching the cracking load, a characteristic abrupt reduction in the slope of the load-deflection curves was observed, marking the transition from the uncracked state to the post-cracking phase.

For the specimens that reached their flexural capacities (R, F-100-2.6, F-100-3.8, and S-50-3.8), the post-cracking stiffness remained approximately constant during the linear-elastic phase of the concrete. However, once the concrete lost its elasticity, the response was characterized by a progressive loss of stiffness, leading to more ductile behaviour. In contrast, specimens that failed prematurely due to shear (F-200-2.6, S-100-2.6, S-200-2.6, S-200-3.8, T-100-2.6, and T-200-2.6) maintained a relatively stable post-cracking stiffness until the point of failure. In these cases, the load-deflection response was governed by the initiation and rapid propagation of a dominant main shear crack. Upon the development of this critical diagonal crack, these specimens exhibited a brittle load drop, indicating a sudden exhaustion of the shear-transfer mechanism. A distinct behavior was noted in specimens F-200-3.8 and S-100-3.8, where a progressive stiffness degradation was recorded prior to failure. This behavior suggests that the evolution of the main shear crack was more gradual, leading to a steady deterioration of the aggregate interlock and the stirrup-concrete bond (particularly in the S-100-3.8 specimen) before the final shear-induced collapse.

### 3.3. Ultimate Load & Energy Absorption Capacities & Deformability Factor

The ultimate load capacities (*P_b_*), the energy absorption capacities (*E_b_*) and the deformability factor (*DF_b_*) of the tested specimens are summarized in Table 3. In this study, the energy absorption capacities (*E_b_*) are defined as the total area under the load-deflection curve up to the point of maximum load. Due to the linear-elastic nature of GFRP reinforcement and the absence of a yielding plateau, traditional ductility indices based on steel yielding are inapplicable to the tested specimens. Consequently, in accordance with ACI 440.1R-15 [4], the structural performance was evaluated through the “deformability factor” (*DF_b_*). According to ACI 440.1R-15 [4], a deflection limit of L/240 was adopted as the reference serviceability limit state (SLS). For the specimens with a/d ratios of 2.6 and 3.8, these SLS thresholds correspond to deflections of 8.33 mm and 11.25 mm, respectively.

The test specimens that have reached their bending capacity—the reference specimens (F-100-3.8 and F-100-2.6)—are the ones within their respective experimental groups with the highest load-carrying capacity and energy absorption capacity. However, for the R specimen, each of its load and energy absorption capacities is approximately 9% higher, while its deformability factor is approximately 13% higher than those of F-100-3.8. The main reason for this is the higher confinement effect of the steel stirrup in the constant moment region compared to the GFRP F-type stirrup, and the limitation that GFRP stirrups cannot be bent at a 135° hook angle. Furthermore, the S-50-3.8 test specimen, which is part of the first experimental group, exhibits a load capacity approximately 7% lower, an energy absorption capacity and a deformability factor value approximately 3% lower than those of F-100-3.8. This suggests that at a reduced spacing (s = 50 mm), the S-type stirrup configuration effectively replicates the behavior of factory-fabricated closed stirrups by providing a stable shear-transfer mechanism.

Conversely, specimens that failed prematurely due to shear exhibited a dramatic reduction in energy dissipation performance. For instance, specimens S-200-3.8 and T-200-2.6 showed *E_b_*/*E_reference_* ratios as low as 0.13 and 0.09, respectively; similarly, their *DF_b_*/*DF_reference_* ratios were found to be 0.15 and 0.09, respectively. This abrupt decline in capacity is a direct consequence of the brittle nature of diagonal tension shear failure. In these cases, the rapid propagation of the main shear crack leads to a premature exhaustion of the aggregate interlock and stirrup-concrete bond. These findings underscore that while alternative stirrup configurations can provide adequate strength, their efficiency in maintaining energy absorption is highly sensitive to reinforcement spacing and the mechanical anchorage integrity of the stirrup-longitudinal bar interface.

### 3.4. Evaluation of Parameters

#### 3.4.1. GFRP Stirrup Type

**Figure 9:**
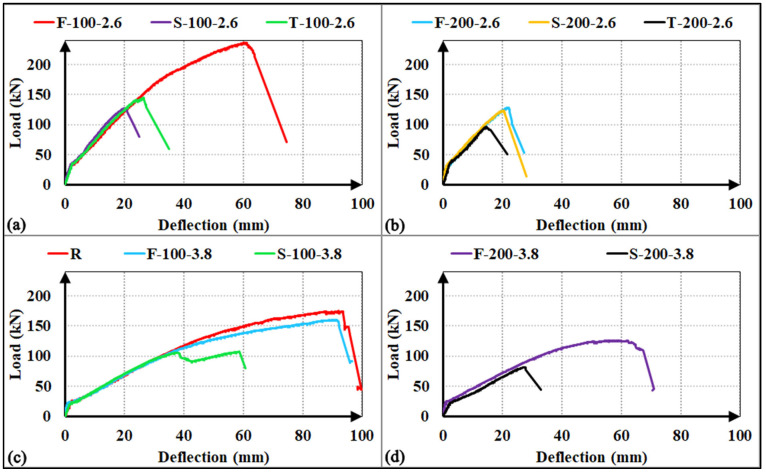
Load–deflection curves of the RC beams for observing the effect of stirrup types: (**a**) shear span-to-depth ratio a/d = 2.6, s = 100 mm; (**b**) a/d = 2.6, s = 200 mm; (**c**) a/d = 3.8, s = 100 mm; (**d**) a/d = 3.8, s = 200 mm.

**Figure 10:**
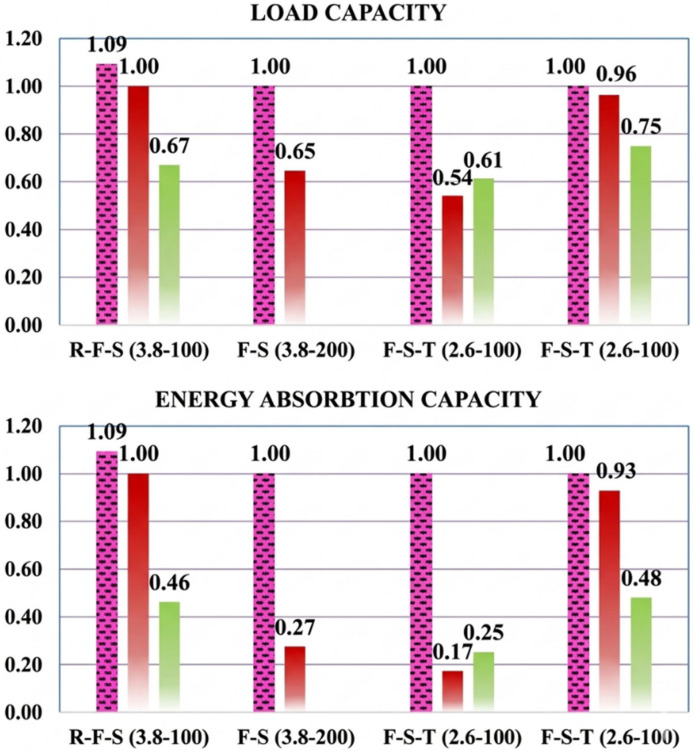
Relative load and energy absorption capacities of the specimens (for evaluation of stirrup types).

The load-deflection responses and relative performance metrics illustrated in Figure 9 and Figure 10 indicate that the factory-fabricated F-type stirrups consistently establish the upper bound for both load-carrying capacity and energy absorption. Transitioning from F-type to site-integrated S-type or T-type configurations typically resulted in a performance decline, primarily driven by a shift in the failure mechanism from relative ductile flexure to brittle shear. For the a/d = 3.8 series, the S-100-3.8 specimen exhibited a 33% reduction in ultimate load and a 54% decrease in energy absorption compared to the F-100-3.8, while the S-200-3.8 showed even more drastic losses (35% and 73%, respectively). This significant drop in energy dissipation is attributed to the low shear strength of the S-type assembly at wider spacings and the inability of discrete bars to maintain the post-cracking stiffness observed in the F-type specimens. In the more shear-critical a/d = 2.6 series, these variations were further amplified; relative to the flexure-governed F-100-2.6, the S-100-2.6 and T-100-2.6 specimens recorded sharp energy absorption drops of 83% and 75%, respectively, as a direct consequence of the brittle shear collapse.

A notable exception was observed at the 200 mm spacing (a/d = 2.6), where the performance gap between S-200-2.6 and F-200-2.6 was limited to approximately 5%. This localized convergence stems from the premature rupture of the F-type stirrups caused by stress concentrations at the bend zones, which prevented the specimen from utilizing its capacity. Finally, the comparative performance between S-type and T-type configurations—where T-type performed better at 100 mm and S-type at 200 mm—suggests that both systems provide a similar degree of structural confinement integrity. These minor fluctuations likely arise from stochastic variations in local concrete properties rather than a fundamental mechanical difference between the two site-integrated systems, indicating that they offer comparable contributions to shear resistance.

Unlike F-type stirrups, the proposed S-Type and T-Type configurations can be easily produced from straight reinforcement bars. These bars can be cut to the required lengths on-site using angle grinders and assembled using conventional tie wires. This practical approach was indeed a primary motivation for investigating alternative stirrup types, as it addresses the logistical challenges of factory-bent GFRP. By enabling a modular installation within the reinforcement matrix, the process is significantly simplified, reducing labor time and the risk of fiber damage. Furthermore, utilizing straight segments instead of sharp factory bends circumvents the ‘bend effect’—the localized strength reduction at corners—thereby enhancing the constructability of the reinforcement system.

#### 3.4.2. GFRP Stirrup Spacing

**Figure 11:**
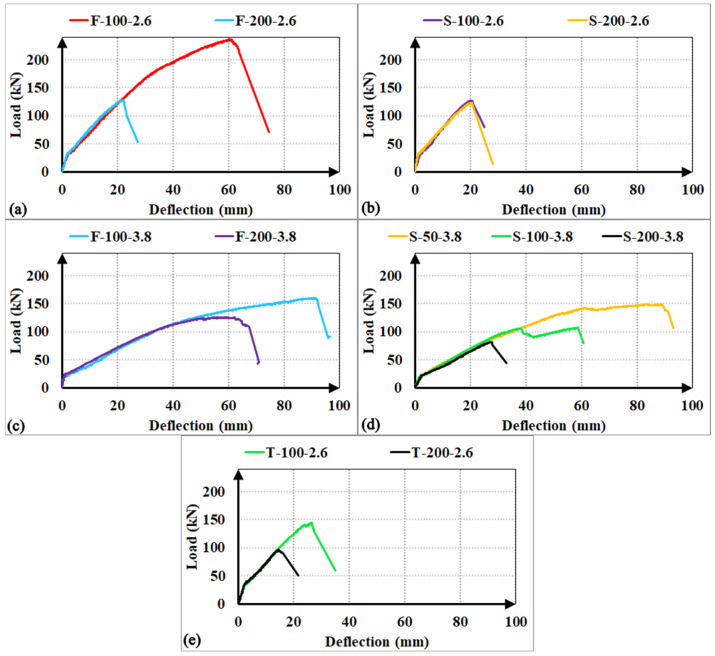
Load–deflection curves of the RC beams for observing the parameter ‘stirrup spacing’: (**a**) shear span-to-depth ratio a/d = 2.6 with F-type stirrups; (**b**) a/d = 2.6 with S-type stirrups; (**c**) a/d = 3.8 with F-type stirrups; (**d**) a/d = 3.8 with S-type stirrups; (**e**) a/d = 2.6 with T-type stirrups.

**Figure 12:**
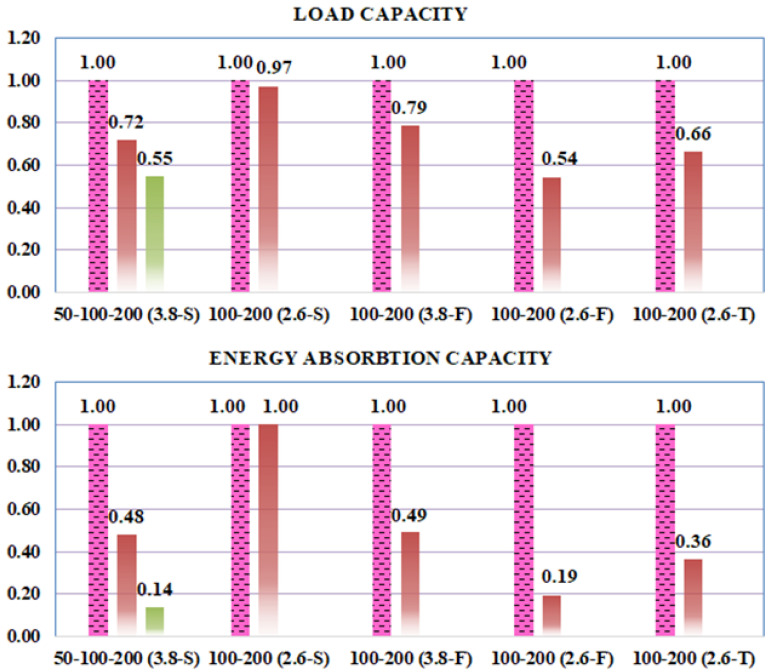
Relative load and energy absorption capacities of the specimens (for evaluation of stirrup spacing).

The comparative load-deflection responses and relative performance metrics illustrated in Figure 11 and Figure 12 demonstrate that increasing the GFRP stirrup spacing consistently triggers a transition in failure mechanisms, leading to a marked degradation in structural performance. For the F-type stirrups, doubling the spacing from 100 mm to 200 mm induced a shift from flexural to brittle shear failure, resulting in significant load capacity reductions of 21% for the F-200-3.8 specimen and 46% for the F-200-2.6 specimen. This decline was accompanied by substantial losses in energy absorption, with the F-200-3.8 showing a 51% decrease and the F-200-2.6 experiencing a sharp 81% decrease. The latter’s severe degradation is primarily attributed to the premature and unexpected rupture of the GFRP stirrup at the localized bend zones. Similarly, the S-type specimens exhibited high sensitivity to reinforcement density; while close stirrup placement (s = 50 mm) successfully facilitated a flexural failure mode, increasing the interval to 100 mm and 200 mm for the a/d = 3.8 group led to load capacity drops of 28% and 45% (S-100-3.8 and S-200-3.8), with corresponding energy absorption collapses of 52% and 86%. Conversely, in the S-type a/d = 2.6 series, the load capacity of the S-200-2.6 specimen decreased by a negligible 3% compared to its 100 mm counterpart, suggesting that at lower a/d ratios, the capacity is governed more by stochastic concrete variations than by reinforcement spacing. In the case of T-type stirrups, increasing the spacing to 200 mm for the a/d = 2.6 series resulted in a 34% decrease in load capacity and a 64% drop in energy dissipation without altering the shear-dominant failure mode. In summary, these findings demonstrate that while site-integrated stirrups provide a viable alternative in terms of ultimate strength, their structural efficiency and ability to sustain integrity are strictly governed by the optimization of stirrup spacing to mitigate the risk of brittle shear failure.

#### 3.4.3. a/d Ratio

**Figure 13:**
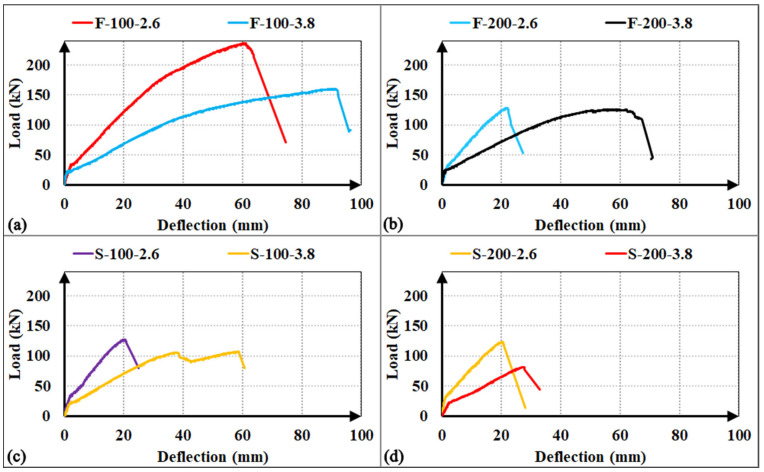
Load–deflection curves of the RC beams for observing the parameter ‘a/d ratio’: (**a**) F-type stirrups with spacing s = 100 mm; (**b**) F-type stirrups with s = 200 mm; (**c**) S-type stirrups with s = 100 mm; (**d**) S-type stirrups with s = 200 mm.

The structural behaviour as a function of the a/d ratio is illustrated through the comparative load-deflection profiles in Figure 13, where specimens are grouped by identical stirrup configuration and spacing. Since all tested specimens maintained an a/d ratio greater than 2.5, their behavior is strictly governed by ‘beam action’ mechanisms, as the slenderness prevents the development of ‘arch action’. Within this slender beam regime, variations in the a/d ratio primarily influenced the shear demand rather than shifting the fundamental failure modes.

Notably, specimens with F-type stirrups at 100 mm spacing reached their ultimate flexural capacities, limiting a direct comparative assessment of their absolute shear strength. However, a consistent trend of higher peak load resistance was observed in the remaining specimens as the a/d ratio decreased from 3.8 to 2.6. Specifically, the a/d = 2.6 series exhibited apparent shear strength increments of approximately 19% for the S-type specimens at 100 mm spacing and up to 51% for the S-type configurations at 200 mm spacing. These improvements are more accurately interpreted as the reinforcement system’s ability to withstand the higher shear demand required to reach flexural limits at lower a/d ratios. The S and T configurations demonstrated sufficient shear resistance to accommodate these increased internal forces. Conversely, the F-200-2.6 specimen showed a negligible strength increase of only 2%, which is attributed to the premature rupture of the GFRP stirrup at the bend zones, preventing the beam from meeting the required shear demand.

## 4. Analytical Study

### 4.1. Rewiew of the Codes & Models for Shear Strength

The shear strength of concrete members reinforced with FRP bars is a complex phenomenon governed by several interacting mechanisms, including aggregate interlock, dowel action, and the resistance of the uncracked compression zone. Unlike conventional steel-reinforced sections, the lower elastic modulus of GFRP bars results in wider cracks and a reduced neutral axis depth, which necessitates specialized analytical models. In this study, the total nominal shear capacity (*V_n_* = *V_c_ + V_frp_*) is evaluated as the sum of the concrete contribution (*V_c_*) and the FRP stirrup contribution (*V_frp_*). In this section, the codes and models frequently employed in shear strength (with or without stirrups) calculations have been discussed.

### 4.2. International Design Codes

#### 4.2.1. ACI 440.1R-15 [4]

The ACI 440.1R-15 guideline recommends the use of Equation (1), developed by Tureyen and Frosch [26], to calculate the concrete shear contribution (*V_c_*). In this expression, the axial stiffness of the longitudinal reinforcement is incorporated through the elastic neutral axis depth (*kd*), where *k*, as defined in Equation (2), represents the ratio between the depth of the neutral axis of the cracked transformed section and the tensile reinforcement effective depth (*d*). The modular ratio (*n_f_* = *E_f_/E_c_*) is the ratio of the modulus of elasticity of the FRP bars (*E_f_*) to that of the concrete (*E_c_*), while *ρ_f_* indicates the longitudinal reinforcement ratio. Additionally, *f_c_* represents the compressive strength of concrete and *b_w_* denotes the beam width.

(1) $$V_{c}=0.4\sqrt{f_{c}}b_{w}d\left(kd\right)$$

(2) $$k=\sqrt{2\rho_{f}n_{f}+{\left(\rho_{f}n_{f}\right)}^{2}}-\rho_{f}n_{f}$$

For the shear strength provided by FRP stirrups perpendicular to the axis of the member (*V_frp_*), ACI 440.1R-15 [4] suggests Equations (3) and (4). Here, *A_fv_* is the cross-sectional area of transverse reinforcement, *f_fv_* is the transverse reinforcement stress, *E_fv_* is the modulus of elasticity of the FRP stirrup, and *s* represents the stirrup spacing. To prevent premature failure at the bending points, a limit strain value of 0.004 is recommended.

(3) $$V_{frp}=\frac{A_{fv}f_{fv}d}{s}$$

(4) $$f_{fv}=0.004E_{fv}\leq f_{fuv}$$

#### 4.2.2. CSA S806-12 [2]

In CSA S806-12, *V_c_* is calculated using Equation (5), developed by Razaqpur and Isgor [27]. This formulation incorporates several critical parameters as shown in Equation (6), such as the moment-shear interaction factor (*k_m_*), the longitudinal reinforcement stiffness factor (*k_r_*), the size effect factor (*k_s_*), and the arch action coefficient (*k_a_*). Within these expressions, *ϕ_c_* is the resistance factor for concrete, *λ* accounts for concrete density, while *M_f_* and *V_f_* stand for the factored moment and factored shear, respectively. The value of *k_m_* cannot exceed 1.0, and *k_s_* accounts for the strength decrease in members deeper than 300 mm. The parameter *k_a_* takes into account the enhancing effect of the concrete shear strength in deep RC beams (a/d ≤ 2.5). In other cases (a/d > 2.5), it takes a value of 1.

(5) $$V_{c}=0.05\lambda \phi_{c}k_{m}k_{r}k_{s}k_{a}{\left(f_{c}\right)}^{1/3}b_{w}d_{v}$$

(6) $$\begin{array}{l} k_{m}={\left(\frac{V_{f}}{M_{f}}d\right)}^{1/2}\leq 1\\ k_{r}=1+{\left(\rho_{f}E_{f}\right)}^{1/3}\\ k_{s}=\left(\frac{750}{450+d}\right)\leq 1\\ k_{a}=\left(\frac{2.5V_{f}}{M_{f}}d\right)\leq 1 \end{array}$$

The contribution of FRP stirrups is determined using Equations (7) and (8), where a limit strain of 0.005 is suggested for the bending points. The calculation employs the effective shear depth (*d_v_*), taken as the maximum of 0.9*d* or 0.72*h*, and considers the inclination angle of the main shear crack (*θ*), which varies between 30° and 60° based on the strain of longitudinal reinforcement (*ε_1_*). *N_f_* and *ϕ_f_* represent the factored axial normal load and the resistance factor for FRP, respectively.

(7) $$V_{frp}=\frac{0.4\phi_{F}A_{fv}f_{fv}d_{v}}{s}\cot\theta$$

(8) $$\begin{array}{c} f_{fv}\leq 0.005E_{f}\\ \theta =30{}^{\circ}+7000\epsilon_{1},\;30{}^{\circ}\leq \theta \leq 60{}^{\circ},\;\epsilon_{1}=\frac{M_{f}/d_{v}+V_{f}+0.5N_{f}}{2E_{f}A_{f}} \end{array}$$

#### 4.2.3. ISIS-M03-07 [24]

The ISIS-M03-07 code expresses *V_c_* as a function of the effective depth through Equation (9) to account for the size effect. For the contribution of FRP stirrups (*V_frp_*), the code prescribes Equations (10)–(12), which consider the inclination angle of the critical shear crack. To safeguard against the premature rupture of GFRP stirrups at the bend zones, a conservative limit strain of 0.0025 is recommended. Furthermore, the calculation utilizes an effective shear depth (*d_v_*) taken as 0.9*d*, where *f_fuv_* represents the ultimate tensile strength of the FRP stirrup material.

(9) $$v_{c}=\left\{\begin{array}{ll} 0.2\lambda \phi_{c}\sqrt{f_{c}}b_{w}d\sqrt{\frac{E_{f}}{E_{s}}} & \mathrm{for}\;d\leq 300\;\mathrm{mm}\\ \left(\frac{260}{1000+d}\right)\lambda \phi_{c}\sqrt{f_{c}}b_{w}d\sqrt{\frac{E_{f}}{E_{s}}} & \mathrm{for}\;d>300\;\mathrm{mm} \end{array}\right.$$

The ISIS-M03-07 [24] code prescribes the calculation of shear strength of FRP stirrups, considering the main shear crack inclination angle, through Equations (10)–(12). It is recommended to use a limit strain value of 0.0025 for the stirrup bending point and an effective shear depth of *d_v_* = *0.9d* for shear calculations. *f_fuv_* stands for the ultimate tensile strength of FRP stirrups. Additionally, the axial normal stress (*σ_N_*) was taken as zero in this study since the specimens were subjected to transverse loading only.

(10) $$V_{frp}=\frac{A_{fv}f_{fv}d_{v}\cot\theta }{s}$$

(11) $$f_{fv}=\frac{(0.05r_{b}/d_{b}+0.3)f_{fuv}}{1.5}\;\mathrm{or}\;\;\;f_{fv}=E_{fv}\epsilon_{fv}$$

(12) $$\epsilon_{fv}=0.0001\sqrt{f_{c}\frac{\rho_{f}E_{f}}{\rho_{fv}E_{fv}}}\left[1+2\left(\frac{\sigma_{N}}{f_{c}}\right)\right]\leq 0.0025$$

#### 4.2.4. CNR DT203-06 [25]

The Italian guideline CNR DT203-06 adopts Equation (13) for evaluating *V_c_*, applicable provided that *ρ_f_* does not exceed 0.02. The formulation introduces a size effect factor (*k_d_*), determined via Equation (14), based on the principle that shear strength decreases as the section depth increases. The design shear stress (*τ_r_*) is formulated in Equation (15) as a function of the characteristic tensile strength of concrete (*f_t_*), while the modular ratio is adjusted using the elasticity modulus of steel (*E_s_* = 200 GPa). In addition, the expression for $1.3{\left(E_{f}/E_{s}\right)}^{1/2}\leq 1$ should not exceed a value of 1. Regarding *V_frp_*, the code mitigates the risk of bend-point failure through Equation (16) by applying a substantial safety factor, effectively limiting the design tensile strength of the stirrup’s straight portion to half of its ultimate capacity (*f_fv_*). In the analytical predictions, the axial normal stress (*σ_N_*) acting on the cross-section was considered to be zero, consistent with the experimental boundary conditions.

(13) $$v_{c}=1.3{\left(\frac{E_{f}}{E_{s}}\right)}^{1/2}\tau_{r}k_{d}(1.2+40\rho_{f})b_{w}d$$

(14) $$k_{d}=1.6-\frac{d}{1000}\geq 1$$

(15) $$\tau_{r}=0.25f_{t}$$

(16) $$V_{frp}=\frac{A_{fv}f_{fv}d}{s}$$

### 4.3. Models

In this section, commonly used models for the shear strength of FRP RC beams without stirrups are provided. In all of these models, the axial rigidity of longitudinal reinforcement and the a/d ratio are taken into consideration for the concrete contribution.

#### 4.3.1. Nehdi et al. [28]

Nehdi et al. proposed a new expression, denoted as Equation (17), to estimate *V_c_*. This model was developed using genetic programming based on a database of 68 experimental tests conducted on FRP-reinforced concrete beams without transverse reinforcement.

(17) $$v_{c}=\left\{\begin{array}{ll} 2.1{\left[f_{c}\rho_{f}\frac{E_{f}}{E_{s}}\frac{d}{a}\right]}^{0.3}b_{w}d & \mathrm{for}\;\frac{a}{d}\geq 2.5\\ 2.1{\left[f_{c}\rho_{f}\frac{E_{f}}{E_{s}}\frac{d}{a}\right]}^{0.3}b_{w}d\times \frac{2.5d}{a} & \mathrm{for}\;\frac{a}{d}<2.5 \end{array}\right.$$

#### 4.3.2. Kara [29]

Kara developed a semi-analytical expression, presented in Equation (18), utilizing an experimental database comprising 110 beams. The approach focuses on capturing the transition of shear transfer mechanisms in FRP-RC members, ensuring that the lower modular ratio of FRP bars is adequately reflected in the concrete’s shear resistance.

(18) $$V_{c}={\left[6.8\sqrt[{3}]{\frac{d}{a}f_{c}\rho_{f}\frac{E_{f}}{E_{s}}}\right]}^{1/3}b_{w}d$$

#### 4.3.3. Ali et al. [1]

Ali et al. proposed a comprehensive model in Equation (19), derived from a large-scale analysis of 510 experimental data points using multivariable nonlinear regression. This model introduces specialized coefficients to account for the physical boundary conditions of the member. The section shape factor (*λ_sh_*) is utilized to distinguish between different geometries, with recommended values of 1.0 for rectangular sections and 0.75 for circular sections. Furthermore, the model incorporates a shear mechanism factor (*λ_a_*) to account for the arch action effect. For members where the a/d ratio is greater than 2.5, *λ_a_* is taken as 1.0, representing standard beam action. Conversely, for beams with an a/d ratio less than 2.5, *λ_a_* is defined as 1/3, reflecting the enhanced capacity and stress redistribution characteristic of deep beam behavior.

Ali et al. [1] proposed Equation (19) for the shear strength of concrete based on 510 experimental data using multivariable nonlinear regression analysis.

(19) $$V_{c}=0.35\lambda_{sh}{\left(E_{f}\rho_{f}\right)}^{1/4}{\left(f_{c}\right)}^{1/4}{\left(\frac{1}{1+0.005d}\right)}^{1/2}{\left(\frac{d}{a}\right)}^{\lambda_{a}}b_{w}d$$

### 4.4. Evaluation of Shear Capacity Predictions

**Figure 14:**
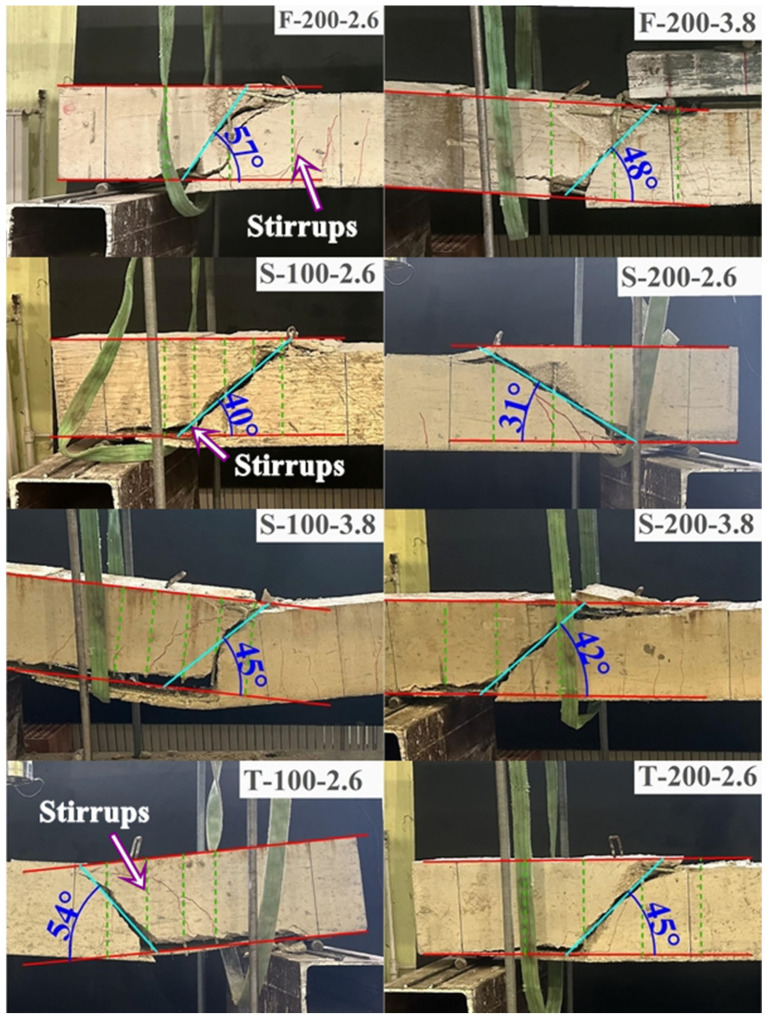
The inclination angle of the main shear crack was obtained from tests.

In Table 4, the shear strengths of FRP RC beams failed by shear failure (*V_n_ = V_c_ + V_frp_*) have been estimated using different codes and compared with experimental data (*V_exp_*). The limit strain value, which takes into account the shear rupture from the bending point, has been used for the shear strength of FRP stirrups in each code. In the calculations performed using CSA S806-12 [2] and ISIS M03-07 [24] codes, the inclination angle of the main shear crack was obtained from experimental data (Figure 14). Furthermore, all material coefficients have been assumed to be 1.

For the specimens utilizing conventional F-type stirrups (F-200-3.8 and F-200-2.6), ACI 440.1R-15 [4] and CSA S806-12 [2] provided conservative estimates, with ACI 440.1R-15 [4] yielding the most accurate predictions (*V_exp_/V_n_* of 1.02 and 1.04), whereas ISIS M03-07 [24] and CNR DT203-06 [25] resulted in non-conservative overestimations. In the case of S-type and T-type stirrup specimens, the CSA S806-12 [2] code, which utilized experimental crack inclination angles (*θ*) ranging from 31° to 57°, as shown in Figure 14, emerged as the most consistent predictor with a mean *V_exp_/V_n_* of 0.81 and a COV of 14.38%. Conversely, CNR DT203-06 [25] exhibited the most significant discrepancy (Mean = 0.35, COV = 24.09%), primarily due to a substantial overestimation of the stirrup contribution in these innovative configurations. The general trend of higher analytical predictions compared to experimental data for S- and T-type specimens indicates that these configurations are less effective than F-type stirrups, implying lower strain engagement and a potential reduction in the overall efficiency of the shear reinforcement.

### 4.5. Evaluation of Transverse Reinforcement Strain and Stirrup Efficiency

In the analytical prediction of the shear strength of FRP RC beams, the total resistance is fundamentally decomposed into the concrete contribution (*V_c_*) and the transverse reinforcement contribution (*V_frp_*). A critical challenge in this quantification is that GFRP-fabricated stirrups often exhibit premature failure at bending points due to localized stress concentrations, leading to lower effective strain values compared to longitudinal bars. While international design codes prescribe various limit strain values—specifically 0.005 in CSA S806-12 [2], 0.004 in ACI 440.1R-15 [4], and a more conservative 0.0025 in ISIS-M03-07 [24]—literature suggests these limits often incorporate excessive safety margins, complicating the precise differentiation between concrete and stirrup contributions.

To isolate the experimental contribution of GFRP stirrups, this study first established a method for *V_c_* by reviewing established models for beams with a/d ratios greater than 2.5. Analyses of experimental data reveal varying degrees of predictive accuracy; for instance, Ali et al. [1] reported average *V_exp_/V_c_* ratios of 1.07 for CSA S806-12 [2] and a highly conservative 2.03 for ACI 440.1R-15 [4], while their proposed model achieved a ratio of 1.05. Similarly, El Zareef et al. [11] analyzed 386 datasets and determined average *V_exp_/V_c_* of 1.1 for CSA S806-12 [2], 1.9 for ACI 440.1R-15 [4], and 0.9 for CNR DT203-06 [25], whereas Alguhi and Tomlinson [12] evaluated 147 GFRP-reinforced beams, noting ratios of 1.04 for CSA S806-12 [2], 1.96 for ACI 440.1R-15 [4], 1.11 for CNR DT203-06 [25], and 1.38 for ISIS-M03-07 [24]. For members with *d_v_* < 300 mm, the average *V_exp_/V_c_* ratios were reported as 1.03 for CSA S806-12 [2], 1.89 for ACI 440.1R-15 [4], and 1.02 for CNR DT203-06 [25]. Furthermore, Alam and Gazder [13], using 196 test data points, confirmed the consistency of CSA S806-12 [2] with an average ratio of 1.06, while the model developed by Kara [29] yielded a precise average of 1.03 across 104 experimental data points.

To establish a scientifically robust baseline for the concrete shear contribution, this study integrates the statistical findings of Kartal [30]. Based on a comprehensive analysis of 149 experimental data points for FRP beams without stirrups, specific *V_exp_/V_c_* ratios were identified as 1.17 (CSA S806-12 [2]), 1.02 (CNR DT203-06 [25]), 1.04 (Kara [29]), and 1.10 (Ali et al. [1]). To align theoretical predictions with experimental observations, a Model Precision Coefficient (*γ_k_*) is introduced. The actual concrete shear contribution (*V_c,act_*) is thus defined as:

(20) $$V_{c,act}=\gamma_{k}\cdot V_{c,p}$$

where *V_c,p_* represents the predicted concrete shear strength according to the respective design code or analytical model. This calibration is instrumental in isolating the net shear force (*V_f,act_*) resisted by the GFRP stirrups (Equation (21)), thereby providing a scientifically robust framework for evaluating the nominal strain in the proposed site-integrated S-type and T-type reinforcement systems. Once *V_f,act_* is determined, the nominal strain of the GFRP stirrups (*ε_fv,n_*) can be derived using Equation (22).

(21) $$V_{f,act}=V_{\exp}-V_{c,act}$$

(22) $$\varepsilon_{fv,n}=\frac{s\cdot V_{f,act}}{A_{fv}\cdot d\cdot E_{fv}}$$

As presented in Table 4, a critical discrepancy was observed as all evaluated design codes provided non-conservative (overestimated) shear capacity predictions for S-type and T-type specimens. The CNR DT203-06 [25] model yielded the most significant overestimation with a mean *V_exp_/V_n_* of 0.35, while CSA S806-12 [2] emerged as the most consistent predictor with a ratio of 0.81. These analytical predictions were conducted by strictly adhering to the code-recommended strain limits for the bend zones of GFRP stirrups. Despite incorporating these conservative strain limits—intended to account for the ‘bend effect’ in traditional closed loops—the codes still substantially overestimated the actual capacity of S and T-type configurations. This systematic overestimation indicates that the non-conservative nature of the codes is not merely a result of material strength assumptions, but rather a fundamental inability to capture the reduced efficiency of discrete, site-integrated reinforcement matrices. From a theoretical perspective, this behavior can be interpreted through the modified truss analogy, where the shear resistance is governed by the interaction between inclined concrete compression struts and transverse tension ties.

As presented in Table 5, the nominal strain values of GFRP stirrups demonstrate that reinforcement efficiency is highly dependent on both stirrup type and geometric constraints. Notably, the highest nominal strain values across all evaluated models were consistently obtained for F-type stirrups, particularly in those specimens with reduced stirrup spacing. Specimen F-100-2.6 attained its theoretical flexural capacity with a peak nominal strain of 0.0044 (CNR DT203-06 [25]), reflecting a significant escalation from the 0.0023 measured in F-100-3.8. This increase is a direct consequence of the reduction in the a/d ratio, which steepens internal stress paths and imposes higher demands on the stirrups to maintain the compression strut’s integrity. While a dense reinforcement layout effectively mitigated premature failures under these conditions, this stability was not sustained at wider intervals; specimen F-200-2.6* underwent rupture at the bend zone at a markedly lower strain of 0.0011 (CSA S806-12 [2]).

Conversely, the S and T-type stirrups generally exhibited lower strain values compared to their F-type counterparts, suggesting a distinct structural activation characteristic. These innovative site-integrated stirrups did not reach high-stress states as rapidly, a phenomenon clearly evidenced in the performance of specimen S-50-3.8. Despite having the most intensive reinforcement ratio (50 mm spacing) in the experimental program, S-50-3.8 attained its theoretical flexural capacity while maintaining remarkably low nominal strain levels of 0.0010 (CNR DT203-06 [25]) and 0.0008 (CSA S806-12 [2]/Kara [29]). This behavior indicates that S-types facilitate a highly efficient load-transfer mechanism that ensures structural stability without necessitating extreme strain accumulation. The discrete nature of S and T-type stirrups introduces a strain lag in the tension tie activation compared to the rigid, continuous box action of conventional F-type loops. In F-type stirrups, the closed geometry facilitates rapid stress mobilization, leading to higher strain engagement (e.g., 0.0044 in F-100-2.6). In contrast, S and T-type stirrups exhibit lower strain values (e.g., 0.0008–0.0010 in S-50-3.8), suggesting a shift in the shear transfer mechanism. In these modular systems, a larger proportion of the internal shear demand is initially sustained by the concrete matrix through aggregate interlock and dowel action. This “load-sharing” mechanism prevents localized stress concentrations at reinforcement nodes, allowing the beam to achieve its ultimate capacity through a more uniform stress distribution across the shear plane. Consequently, the modular S and T-type configurations avoid sudden shear-compression failure by maintaining the integrity of the concrete compression strut even at lower reinforcement activation levels.

By eliminating these pre-bent nodes, S and T-type configurations allow internal stresses to spread more evenly along the entire length of the reinforcement. This uniform stress distribution prevents localized pressure build-up, enabling the beams to avoid sudden shear failure even when the stirrups are only minimally active. The superior integrity of these types is further validated by the fact that several S and T-type specimens (marked with * in Table 5) reached their full capacity, with S-200-2.6 maintaining stability at a strain of 0.0027 (CNR DT203-06 [25]).

The analytical evaluation of the selected formulations reveals significant discrepancies in capturing the actual stress development within GFRP stirrups. As evidenced in Table 5, the discrepancy between predictive frameworks is primarily governed by *γ_k_*, which dictates how shear resistance is partitioned between the concrete matrix and the reinforcement. In models prioritizing high safety margins, such as CSA S806-12 [2] and the Ali et al. [1] formulations, a higher *γ_k_* is utilized. This calibration over-attributes shear resistance to the concrete matrix to account for uncertainties in aggregate interlock and dowel action. However, this disproportionate assignment often masks the actual demand on the reinforcement. This is mathematically evident in specimens S-200-3.8 and T-200-2.6, where these models yield near-zero or negative nominal strains (reaching -0.0016). Rather than representing a physical state, these values reflect a numerical offset caused by the mismatch between code-based truss models—calibrated for continuous stirrups—and the experimental reality of modular stirrup engagement. In contrast, the CNR DT203-06 [25] and Kara [29] models employ a more balanced calibration of the concrete contribution. By more accurately reflecting observed failure modes, these frameworks enable a transparent evaluation of load-sharing in innovative stirrup designs.

## 5. Conclusions

The present study offered a detailed experimental and analytical investigation into the shear behavior of reinforced concrete beams using glass fiber-reinforced polymer stirrups, with particular attention to stirrup type, spacing, and shear span-to-depth ratios. The findings provide significant insights into the performance, design, and structural behavior of GFRP-reinforced beams under shear forces, highlighting both the potential and limitations of this reinforcement type.

The investigation demonstrated that fabricated GFRP stirrups exhibit superior structural behavior compared to site-integrated configurations, primarily due to their inherent geometric continuity. Rather than merely increasing load-carrying capacity, these continuous closed-loop stirrups provide more effective concrete confinement. This structural advantage allowed specimens with fabricated stirrups to reach their theoretical flexural capacities even with lower transverse reinforcement ratios. In contrast, the efficiency of site-integrated stirrups was found to be more dependent on transverse reinforcement density, highlighting the critical role of stirrup configuration in maintaining structural reliability.

Stirrup type and spacing emerged as decisive factors in determining failure mechanisms. The study revealed that increasing the spacing of fabricated stirrups or substituting them with site-integrated configurations at similar spacings could shift the failure mode from ductile flexural-dominated behavior to brittle shear-dominated behavior. This finding emphasizes that stirrup characteristics are as influential as the overall reinforcement ratio in maintaining structural integrity. In this context, careful attention to stirrup type and placement is essential to prevent premature shear failures, particularly in beams with low shear span-to-depth ratios.

The experimental results also highlighted differences in energy absorption and peak load between GFRP and steel stirrups. While fabricated GFRP stirrups allowed beams to approach the flexural capacity of steel-reinforced references, their energy absorption and peak load were slightly lower. This difference reflects the inherent benefits of steel stirrups, including superior confinement and dowel action. Nevertheless, fabricated GFRP stirrups provided a more uniform and predictable structural response when free of local defects, whereas site-integrated stirrups were more prone to bond-slip problems and early shear-tension failures.

Beyond mechanical limits, the practical field applicability and economic feasibility of these reinforcement systems are crucial. While factory-fabricated GFRP stirrups offer enhanced structural behavior due to their geometric continuity, their widespread adoption is often hindered by high specialized mold costs required for each specific cross-sectional dimension and the structural vulnerability of bend zones, which are prone to localized stress concentrations. Conversely, site-integrated GFRP stirrups introduce a modular approach that eliminates the need for dimension-specific molds and significantly enhances construction convenience through easy on-site application. By reducing production complexity and allowing for flexible adjustments regardless of beam size, these modular configurations offer a cost-effective alternative, providing a balanced trade-off between overall structural behavior and practical engineering economy.

The hierarchy of parametric influence on shear capacity is dominated by the interdependent relationship between stirrup configuration and spacing. While the shear span-to-depth ratio acts as the primary driver of shear demand, the stirrup spacing functions as the critical switch for failure modes. Specifically, factory-fabricated F-type stirrups establish a performance threshold at 100 mm, whereas site-integrated S-type configurations necessitate a 50 mm interval to successfully reach the ultimate flexural capacity.

Strain development in the transverse reinforcement was shown to be a complex function of both stirrup type and shear span-to-depth ratio. In beams with site-integrated stirrups, lower span-to-depth ratios increased stirrup strain due to steeper internal shear paths. Conversely, in widely spaced fabricated stirrups, strain reductions were observed at lower span-to-depth ratios, attributed to localized rupture at bend zones rather than reduced demand. Peak stirrup strain correlated closely with the attainment of theoretical flexural capacities, confirming the essential role of transverse reinforcement in overall beam performance.

Analytical evaluations demonstrated that certain models provided accurate predictions of shear behavior and stirrup strain, particularly for fabricated stirrups. Models capturing both concrete contribution and stirrup engagement offered reliable assessments of shear capacity and strain distribution, enabling a more precise understanding of failure mechanisms. These findings suggest that properly calibrated analytical tools can serve as effective design aids for GFRP-reinforced beams, supporting safer and more efficient structural applications.

The study confirms that fabricated GFRP stirrups, when properly designed and installed, can deliver reliable and predictable shear performance, although careful consideration must be given to spacing, span-to-depth ratios, and potential local defects. The combination of experimental data and analytical models provides a solid foundation for optimizing GFRP shear reinforcement, enhancing both structural safety and material efficiency. These insights contribute valuable guidance for the design, application, and further development of GFRP-reinforced concrete members.

## 6. Future Perspectives

While the current investigation successfully validates the fundamental shear performance and logistical viability of the proposed S-Type and T-Type GFRP configurations, it is essential to acknowledge certain limitations inherent in the experimental scope. The testing of 12 full-scale RC beams provides a robust structural benchmark; however, it is recognized that this sample size may not be sufficient to achieve full statistical saturation or to establish definitive, generalized design formulations across the entire spectrum of structural scales and environmental conditions. To transition from this pioneer validation toward international code integration (e.g., ACI 440, CSA S806), future research must expand the experimental database to reach the statistical reliability required for definitive empirical modeling. Key research trajectories include evaluating site-integrated configurations in larger structural members with complex geometries to further refine the understanding of the size effect and investigating long-term durability under environmental fatigue. Additionally, while the stiffness and energy dissipation capacity are similar at SLS across the studied groups, acknowledging the potential for stiffness degradation under cyclic or long-term effects remains a distinct and vital parameter. Therefore, investigating the evolution of stiffness and crack width development under sustained loading is recommended to further refine the serviceability limit state equations for these architectures. Furthermore, assessing the energy dissipation and bond-slip behavior of these architectures under cyclic loading remains essential for their application in seismic zones. Ultimately, this work serves as a scientific catalyst, providing the necessary empirical motivation to encourage extensive future research aimed at optimizing high-performance, site-assembled GFRP shear systems.

## Figures and Tables

**Table 1:** Test Specimen Details.

Beam	Stirrup Type	Spacing (mm)	a/d Ratio
R	Steel	100	3.80
F-100-3.8	F-Type (fabricated)	100	3.80
F-200-3.8	F-Type (fabricated)	200	3.80
F-100-2.6	F-Type (fabricated)	100	2.60
F-200-2.6	F-Type (fabricated)	200	2.60
S-50-3.8	S-Type (Integrated-4 straight rebars)	50	3.80
S-100-3.8	S-Type (Integrated-4 straight rebars)	100	3.80
S-200-3.8	S-Type (Integrated-4 straight rebars)	200	3.80
S-100-2.6	S-Type (Integrated-4 straight rebars)	100	2.60
S-200-2.6	S-Type (Integrated-4 straight rebars)	200	2.60
T-100-2.6	T-Type (2 straight vertical rebars)	100	2.60
T-200-2.6	T-Type (2 straight vertical rebars)	200	2.60

**Table 2:** Mechanical properties of GFRP rebars.

Diameter (mm)	Modulus of Elasticity (GPa)	Tensile Strength (MPa)	Ultimate Tensile Strain
Ø12	55	924	0.017
Ø10	42	787	0.019

**Table 3:** Load-capacity and energy absorption capacities of the test specimens.

Group	Beam	*P_b_* (kN)	*E_b_* (kJ)	*DF_b_*	*P_b_/P_reference_*	*E_b_/E_reference_*	*DF_b_/DF_reference_*
1st	R	174.72	10.65	32.27	1.09	1.09	1.13
	F-100-3.8 *	159.79	9.74	28.65	1.00	1.00	1.00
	F-200-3.8	126.07	4.77	12.23	0.79	0.49	0.43
	S-50-3.8	148.80	9.41	27.68	0.93	0.97	0.97
	S-100-3.8	107.44	4.52	13.70	0.67	0.46	0.48
	S-200-3.8	81.67	1.31	4.23	0.51	0.13	0.15
2nd	F-100-2.6 *	236.59	9.21	28.78	1.00	1.00	1.00
	F-200-2.6	128.3	1.74	4.97	0.54	0.19	0.17
	S-100-2.6	127.52	1.59	4.54	0.54	0.17	0.16
	S-200-2.6	123.62	1.61	4.13	0.52	0.17	0.14
	T-100-2.6	145.01	2.32	7.25	0.61	0.25	0.25
	T-200-2.6	96.11	0.84	2.55	0.41	0.09	0.09

*P_b_*—Load Capacity of beams. *E_b_*—Energy Absorption Capacities of beams. *DF_b_*—Deformability factor of beams. *P_reference_*—Load capacity of reference beams. *E_reference_*—Energy Absorption Capacities of reference beams. *DF_reference_*—Deformability factor of reference beams. *—Reference beams within each group.

**Table 4:** Shear strength calculations with different codes.

Beam	Failure Mode	*V_exp_* (kN)	ACI 440-1R-15 [3]		CSA S806-12 [2]		ISIS M03-07 [24]		CNR DT203-06 [25]	
			*V_n_* (kN)	*V_exp_/V_n_*	*V_n_* (kN)	*V_exp_/V_n_*	*V_n_* (kN)	*V_exp_/V_n_*	*V_n_* (kN)	*V_exp_/V_n_*
F-200-3.8	Shear tension	63.04	61.92	1.02	53.07	1.19	78.37	0.80	123.53	0.51
F-200-2.6	FRP stirrup rupture	64.15	61.92	1.04	56.80	1.13	65.11	0.99	123.53	0.52
S-100-3.8 *	Adherence failure and shear tension	53.72	99.53	0.54	71.67	0.75	136.49	0.39	211.65	0.25
S-200-3.8 *	Shear tension	40.84	61.92	0.66	56.62	0.72	89.48	0.46	123.53	0.33
S-100-2.6 *	Shear tension	63.76	99.53	0.64	86.14	0.74	156.79	0.41	211.65	0.30
S-200-2.6 *	Shear tension	61.81	61.92	1.00	73.97	0.84	118.74	0.52	123.53	0.50
T-100-2.6 *	Shear tension	72.51	99.53	0.73	70.40	1.03	107.58	0.67	211.65	0.34
T-200-2.6 *	Shear tension	48.06	61.92	0.78	62.73	0.77	83.64	0.57	123.53	0.39
			Average *	0.72	Average *	0.81	Average *	0.50	Average *	0.35
			STD(%) *	15.68	STD(%) *	11.60	STD(%) *	10.78	STD(%) *	8.50
			COV(%) *	21.67	COV(%) *	14.38	COV(%) *	21.37	COV(%) *	24.09

* For S-type and T-type stirrup specimens, the mean, standard deviation, and coefficient of variation have been calculated.

**Table 5:** Nominal strain values of GFRP stirrups (***ε_fv,n_***).

Beam	CNR DT203-06 [25]	CSA S806-12 [2]	Ali et al. [1]	Kara [29]
F-100-3.8	0.0023	0.0019	0.0020	0.0020
F-200-3.8 *	0.0029	0.0020	0.0021	0.0022
F-100-2.6	0.0044	0.0034	0.0029	0.0039
F-200-2.6 *	0.0030	0.0011	0.0001	0.0021
S-50-3.8	0.0010	0.0008	0.0008	0.0008
S-100-3.8 *	0.0009	0.0005	0.0006	0.0006
S-200-3.8 *	0.0005	−0.0004	−0.0002	−0.0002
S-100-2.6 *	0.0015	0.0005	0.0000	0.0010
S-200-2.6 *	0.0027	0.0009	−0.0001	0.0018
T-100-2.6 *	0.0019	0.0010	0.0005	0.0015
T-200-2.6 *	0.0013	−0.0006	−0.0016	0.0004

* specimens reach their capacity with shear failure.

## Data Availability

The original contributions presented in this study are included in the article. Any further inquiries can be directed to the corresponding authors.

## Abbreviations

a/d	Shear span-to-effective depth ratio
*A_fv_*	Cross-sectional area of FRP transverse reinforcement
*b_w_*	Beam web width
CFRP	Carbon Fiber-Reinforced Polymer
CNR-DT 203-06	Italian National Research Council Guideline for FRP Reinforcement
CSA S806-12	Canadian Standard for Design and Construction of Building Structures with FRP
COV	Coefficient of Variation
*d*	Effective depth of beam
*d_v_*	Effective shear depth
*DF_b_*	Deformability factor of beams
*E_b_*	Energy absorption capacity of beam
*E_c_*	Modulus of elasticity of concrete
*E_f_*	Modulus of elasticity of longitudinal FRP reinforcement
*E_fv_*	Modulus of elasticity of FRP stirrup
*E_s_*	Modulus of elasticity of steel
*f_c_*	Compressive strength of concrete
*f_fuv_*	Ultimate tensile strength of FRP stirrup
*f_fv_*	Stress in FRP transverse reinforcement
FRP	Fiber-Reinforced Polymer
GFRP	Glass Fiber-Reinforced Polymer
GRNN	Generalized Regression Neural Network
*h*	Total height of beam
ISIS-M03-07	ISIS Canada Design Manual No. 3 for FRP Reinforcement
*k_a_*	Arch action factor
*k_d_*	Neutral axis depth ratio (ACI)/size effect factor (CNR)
*k_m_*	Moment-shear interaction factor
*k_r_*	Longitudinal reinforcement stiffness factor
*k_s_*	Size effect factor
*M_f_*	Factored bending moment
*n_f_*	Modular ratio (Ef/Ec)
*P_b_*	Ultimate load capacity
RC	Reinforced Concrete
*s*	Stirrup spacing
STD	Standard Deviation
*V_c_*	Concrete contribution to shear strength
*V_c,act_*	Actual concrete shear contribution
*V_c,p_*	Predicted concrete shear contribution
*V_exp_*	Experimental shear strength
*V_f,act_*	Actual shear force resisted by FRP stirrups
*V_frp_*	FRP stirrup contribution to shear strength
*V_n_*	Nominal shear strength (Vc + Vfrp)
*θ*	Inclination angle of main shear crack
*ρ_f_*	Longitudinal reinforcement ratio
*ρ_fb_*	Balanced reinforcement ratio
*σ_N_*	Axial normal stress
*ε_1_*	Longitudinal reinforcement strain
*ε_fv,n_*	Nominal strain of FRP stirrup
*ϕ_c_*	Concrete resistance factor
*ϕ_f_*	FRP resistance factor
*γ_k_*	Model Precision Coefficient
